# Numerical Investigation of the Fracture Properties of Pre-Cracked Monocrystalline/Polycrystalline Graphene Sheets

**DOI:** 10.3390/ma12020263

**Published:** 2019-01-15

**Authors:** Xinliang Li, Jiangang Guo

**Affiliations:** Tianjin Key Laboratory of Modern Engineering Mechanics, School of Mechanical Engineering, Tianjin University, Tianjin 300072, China; lixl@tju.edu.cn (X.L.)

**Keywords:** polycrystalline graphene, fracture toughness, molecular structure mechanics, grain boundary, strain energy per unit area

## Abstract

The fracture properties of pre-cracked monocrystalline/polycrystalline graphene were investigated via a finite element method based on molecular structure mechanics, and the mode I critical stress intensity factor (SIF) was calculated by the Griffith criterion in classical fracture mechanics. For monocrystalline graphene, the size effects of mode I fracture toughness and the influence of crack width on the mode I fracture toughness were investigated. Moreover, it was found that the ratio of crack length to graphene width has a significant influence on the mode I fracture toughness. For polycrystalline graphene, the strain energy per unit area at different positions was calculated, and the initial fracture site (near grain boundary) was deduced from the variation tendency of the strain energy per unit area. In addition, the effects of misorientation angle of the grain boundary (GB) and the distance between the crack tip and GB on mode I fracture toughness were also analyzed. It was found that the mode I fracture toughness increases with increasing misorientation angle. As the distance between the crack tip and GB increases, the mode I fracture toughness first decreases and then tends to stabilize.

## 1. Introduction

Pristine graphene, a two-dimensional material, consists of carbon atoms and possesses excellent electronic, thermal, and mechanical properties [1]; it has been applied in many fields such as nanocomposites [2,3] and nanoelectronics [4,5], etc. Despite its brilliant performance, it has low fracture toughness. Chemical vapor deposition (CVD) is one of the main techniques used to produce graphene. However, the large-sized graphene obtained by the CVD technique includes defects such as grain boundaries (GBs), cracks [6,7,8], etc. Many researchers have reported that GBs introduced by the CVD technique have an impact on the fracture properties of graphene [9,10,11,12,13,14,15]. Therefore, it is very meaningful to study the influence of GBs on the fracture mechanical properties of graphene.

The fracture properties of monocrystalline/polycrystalline graphene have been investigated by experimental and theoretical methods. The GBs (repeating pentagon–heptagon ring) of graphene were observed via transmission electron microscopy [10,11], and nanoindentation experiments showed that GBs could reduce the failure strength of polycrystalline graphene [11,12]. Grantab et al. [13] performed molecular dynamics (MD) simulations to investigate the failure strength of graphene. They reported that the failure strength of polycrystalline graphene was almost the same as that of pristine graphene when the misorientation angle of GB was large. In addition, many researchers have found that the failure strength of polycrystalline graphene is not only related to the misorientation angle of GB (density of defects), but also to the arrangement of defects. Based on MD simulations, Wei et al. [14] reported that GB strength increased with increasing misorientation angle when the defects had a uniform distribution. This tendency has not appeared in any other case. These variations of GB strength could be interpreted as the interaction of the stress fields of adjacent defects. Xu et al. [15] investigated the fracture properties of bicrystalline graphene via molecular structural mechanics. They found that the failure strength strongly relied on the arrangement of defects. Besides studying the strength of graphene, many works have studied the initial fracture site of polycrystalline graphene. Wu et al. [16] used ab initio calculations and MD simulations to show that the fracture always starts from a GB. However, based on MD simulations, Zhang et al. [17] and Yi et al. [18] reported that the initial fracture site may appear at a GB or away from a GB.

The fracture toughness is very important for the stability of materials. For practical engineering, the available strength of graphene relies not on the failure strength which governs the fracture of the carbon bonds, but on the fracture toughness [19]. Hence, the fracture toughness is more important than the failure strength. Aliha et al. [20,21] studied the mode I, mode III, and mixed mode I/III fracture toughness of graphite by experiment and theory. They found that the specimen called Edge Notched Disc Bend was suitable for investigating the fracture toughness of graphite materials. Jung et al. [22] studied the fracture toughness of polycrystalline graphene through MD simulations. They reported that polycrystalline graphene liberated more energy and that the GB could increase the critical energy release rate of graphene. The reason for this was that GB could reduce the stress concentration at the crack tip. Shekhawat et al. [23] proposed the statistical theory of fracture toughness by using large-scale atomic simulations and continuum modelling. They found that the fracture toughness and failure strength had obvious statistical fluctuations and relied on the grain sizes of polycrystalline graphene. Han et al. [24] performed MD simulations investigating the influence of GB on the fracture toughness. They reported that the fracture toughness increased with increasing misorientation angle. In summary, the fracture properties of graphene have been widely studied. However, the initial fracture site of polycrystalline graphene and the effect of the distance between the crack tip and the GB on the fracture toughness have still not been systematically investigated.

In this paper, the molecular structure mechanics [25] and the Griffith criterion are used to study the fracture properties of monocrystalline/polycrystalline graphene. For monocrystalline graphene, the size-dependent mode I fracture toughness and the influence of crack width and the ratio of crack length and graphene width on the mode I fracture toughness are investigated. For polycrystalline graphene, the initial fracture site of graphene is revealed based on the strain energy per unit area. Then, the effects of misorientation angle of the GB and the distance between the crack tip and GB on the mode I fracture toughness are systematically analyzed.

## 2. Models and Methods

### 2.1. Computational Methods

The interatomic potential energy of graphene can be described through the steric potential energy [25]:

(1) $$U=\sum U_{r}+\sum U_{\theta }+\sum U_{\phi }+\sum U_{\omega }+\sum U_{vdw}$$

where U represents the total potential energy, and $U_{r}$, $U_{\theta }$, $U_{\phi }$, and $U_{\omega }$, are the potential energy related to bond stretching, bond angle bending, dihedral angle torsion, and out-of-plane torsion, respectively. $U_{vdw}$ represents the van der Waals interaction. Generally, the van der Waals force can be ignored in covalent systems. Further,

(2) $$U_{r}=\frac{1}{2}k_{r}{\left(r-r_{0}\right)}^{2}=\frac{1}{2}k_{r}{\left(\Delta r\right)}^{2}$$

(3) $$U_{\theta }=\frac{1}{2}k_{\theta }{\left(\theta -\theta_{0}\right)}^{2}=\frac{1}{2}k_{\theta }{\left(\Delta \theta \right)}^{2}$$

(4) $$U_{\tau }=U_{\phi }+U_{\omega }=\frac{1}{2}k_{\tau }{\left(\Delta \phi \right)}^{2}$$

where $U_{\tau }$ represents the sum of potential energy related to torsion; $k_{r}$, $k_{\theta }$, and $k_{\tau }$ are the force constants for bond stretching, bond angle bending, and bond twisting, respectively; and Δr, Δθ, and Δϕ represent increments in the bond length, bond angle, and angle of bond twisting, respectively.

Based on molecular structure mechanics [25], the equivalent beam elements can be used for replacing C–C bonds in graphene, and honeycomb graphene is thought of as a space frame structure. The strain energy of a beam element has an equivalent relationship with the atomic potential energy of graphene. The parameters of a beam element with a circular cross section can be obtained by

(5) $$\frac{EA}{l}=k_{r},\;\frac{EI}{l}=k_{\theta },\frac{GJ}{l}=k_{\tau }$$

where *l* is the length of the beam element; *A* is the area of the cross section; *I* and *J* represent the moments of inertia and polar moment of inertia, respectively; and *E* and *G* are the elastic modulus and shear modulus, respectively. However, in this method, the different parameters can be derived by different force constants, and the in-plane flexural rigidity of the beam element is equal to the out-of-plane flexural rigidity.

In order to eliminate the above problems, the sectional properties of the beam element are derived from modified molecular structural mechanics [26], so the effect of the force constant on the cross-section parameters is avoided. The beam element has different in-plane and out-of-plane flexural rigidities because of a rectangular section. These parameters can be derived as follows [26]:

(6) $$\begin{array}{c} l=0.1421\;\mathrm{nm},\;\;\;\;\;\;A=b\times h,\;\;\;\;\;\;I_{y}=(b\times h^{3})/12\;\\ I_{z}=(b^{3}\times h)/12,\;\;\;\;\;\;J=I_{y}+I_{z},\;\;\;\;\;\;G=E/(2(1+\mu )) \end{array}$$

(7) $$\begin{array}{l} \frac{EA}{l}=731\;\mathrm{nN}/\mathrm{nm},\;\;\;\;\;\;\;\frac{EI_{z}}{l}=0.677\;\mathrm{nN}\cdot \mathrm{nm}\\ \frac{EI_{y}}{l}=0.267\;\mathrm{nN}\cdot \mathrm{nm},\;\;\;\;\;\;\;\frac{GJ}{l}=0.151\;\mathrm{nN}\cdot \mathrm{nm} \end{array}$$

where *b* and *h* are the width and height of the beam element, respectively; $I_{y}$ and $I_{z}$ represent the moments of inertia; and μ is the Poisson ratio. The parameters are listed in Table 1. According to the parameters in Table 1, the Timoshenko beams (beam188 element in ANSYS 12.0) should be adopted for this research.

Further, the nonlinear behavior of materials is very important for the result of simulations. In this research, the nonlinear mechanics behavior of graphene is described by the modified Morse potential [27,28]:

(8) $$U=U_{r}+U_{\theta }$$

(9) $$U_{r}=D_{e}\left[{\left(1-e^{-\beta \left(r-r_{0}\right)}\right)}^{2}-1\right]$$

(10) $$U_{\theta }=\frac{1}{2}k_{\theta }{\left(\theta -\theta_{0}\right)}^{2}\left[1+k_{sexic}{\left(\theta -\theta_{0}\right)}^{4}\right]$$

where r and θ represent the current bond length and neighboring bond angle, respectively. The parameters of the modified Morse potential are listed in Table 2. For the in-plane deformation of graphene, the change in potential energy caused by the bond angle is smaller than that caused by bond stretching [27,28], and the effect of bond angle bending on the potential energy is very small. Therefore, only the bond stretching energy is considered in this research. From the derivative of Equation (9), the tensile force of the C–C bond can be obtained:

(11) $$F=2\beta D_{e}\left[1-e^{-\beta \left(r-r_{0}\right)}\right]e^{-\beta \left(r-r_{0}\right)}.$$

**Figure 1:**
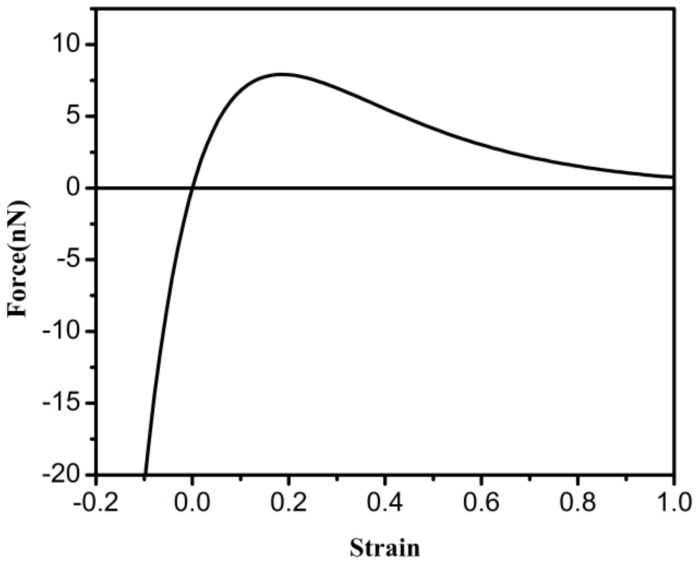
The force–strain curve of C–C bonds.

The variations in force with increasing strain of the C–C bond are exhibited in Figure 1. The strain ε is calculated by $\varepsilon =(r-r_{0})/r_{0}$. The fracture of the bond is mainly governed by the interatomic potential energy before the inflection point, but the potential energy is not important after the inflection point [28]. The equivalent beam element is in equilibrium when the strain is zero. With increasing strain, the inflection point occurs at 19%. The relationship between stress and strain can be derived from the formula σ=F/A and Equation (11).

From the perspective of the strain energy per unit area, the initial fracture site in polycrystalline graphene is now explored. The following equation is proposed for calculating the strain energy per unit area:

(12) $$\sum =\frac{U_{strain}}{m\times n}$$

where ∑ is the strain energy per unit area, *U_strain_* is the total strain energy, and *m* and *n* are the width and length of the graphene nanoribbon, respectively.

The mode I critical stress intensity factor is calculated by the Griffith criterion in classical fracture mechanics [29,30,31]:

(13) $$K=\sqrt{2}D\sigma \sqrt{\pi a}$$

(14) $$D=(1-0.025f^{2}+0.06f^{4})\sqrt{\sec(\frac{\pi f}{2})}$$

(15) $$f=\frac{2a}{W}$$

where *D* is the correction factor, σ is the tensile stress, *2a* is the crack length, *W* is the width of the graphene, and *f* is the proportion of the crack.

### 2.2. Graphene Models

**Figure 2:**
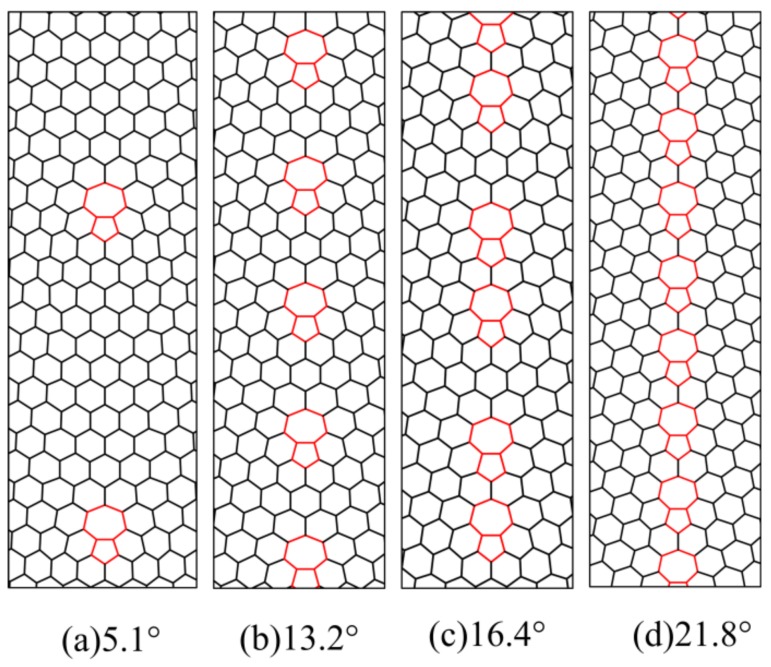
Schematic of the zigzag-oriented grain boundary (GB) models with different misorientation angles.

**Figure 3:**
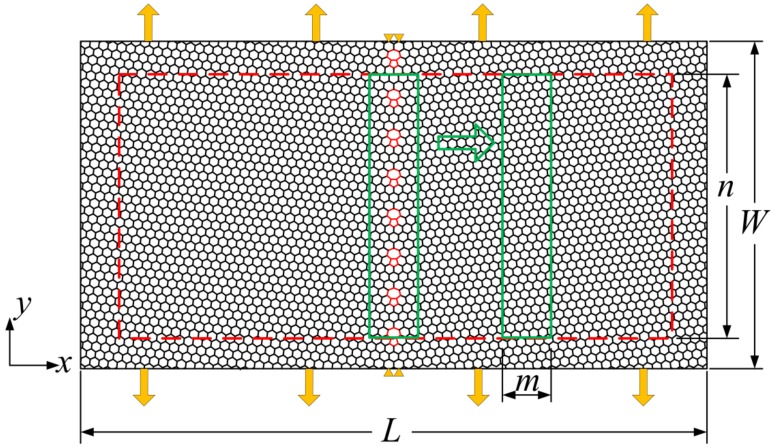
Schematic of the graphene model containing a zigzag-oriented GB.

Zigzag-oriented GB models with different misorientation angles were constructed using molecular structure mechanics and the theory of crystal disclinations, as shown in Figure 2. A detailed definition of the misorientation angle can be found in reference [15]. The GB is composed of pentagon–heptagon ring defects, and the chosen misorientation angles were 5.1°, 13.2°, 16.4°, and 21.8°. A graphene model (as shown in Figure 3) containing a zigzag-oriented GB was fabricated to investigate the initial fracture site of graphene; its size was *L × W* = 17 nm *×* 9 nm, where *L* and *W* denote the length and width of the graphene. The misorientation angles of the GB were 5.1°, 13.2°, 16.4°, and 21.8°. The values of strain energy per unit area of the different graphene nanoribbons were calculated for the area shown in the green solid line frame of Figure 3. In the process of simulations, the green solid line frame of size *n × m* = 8 nm *×* 0.88 nm gradually moves away from the GB. In addition, the movable area of the green solid line frame is confined within the red dotted frame so as to avoid the boundary effect of the graphene. Two types of boundary conditions were adopted for the model in Figure 3. One is that the left and right edges are subjected to opposite displacement load along the *x* direction, and the *y* direction at the middle of the left and right edges is constrained, while the top and bottom edges are free; the other is that the top and bottom edges are subjected to the opposite displacement load along the *y* direction, and the *x* direction at the middle of the top and bottom edges is constrained, while the left and right edges are free, as shown in Figure 3.

**Figure 4:**
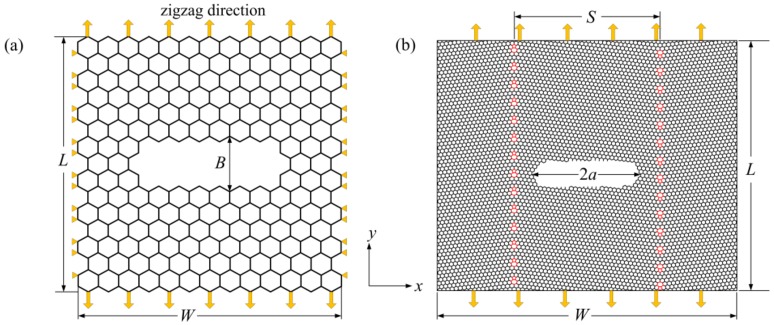
The graphene models containing a central crack: (**a**) zigzag-oriented monocrystalline graphene; (**b**) polycrystalline graphene with two antisymmetric zigzag-oriented GBs.

Monocrystalline/polycrystalline graphene models containing a central crack (perpendicular to the load direction) were created to study the influence of GB on the mode I fracture toughness as shown in Figure 4a,b, where Figure 4a is the zigzag-oriented monocrystalline graphene model and Figure 4b is the square polycrystalline graphene model with two antisymmetric zigzag-oriented GBs. The distance between the two antisymmetric GBs and the crack length and width are represented by *S*, 2*a*, and *B*, respectively. For convenience, the ratio of crack length to graphene width was defined as the crack proportion *f*. In order to simulate the process by which the crack tip gradually expands to the GB, the crack length is kept constant and the distance between the two GBs is gradually reduced. The influence factors can be simplified by this means. The distances between the crack tip and GB were 0.33 nm, 0.6 nm, 0.8 nm, and 1.1 nm. The size of the specimen has a significant influence on the fracture toughness [32]. In this paper, the main object was to study the effect of GBs on the fracture toughness. The size of the polycrystalline graphene model was considered to be constant. In the process of simulation, the misorientation angles of the two antisymmetric GBs were 5.1°, 13.2°, 16.4°, and 21.8°. The same boundary condition was imposed on the two types of graphene models. The top and bottom edges were subjected to opposite displacement load along the *y* direction, while the *x* directions of the left and right edges were constrained, as shown in Figure 4.

## 3. Results and Discussion

### 3.1. The Initial Fracture Site of Polycrystalline Graphene

**Figure 5:**
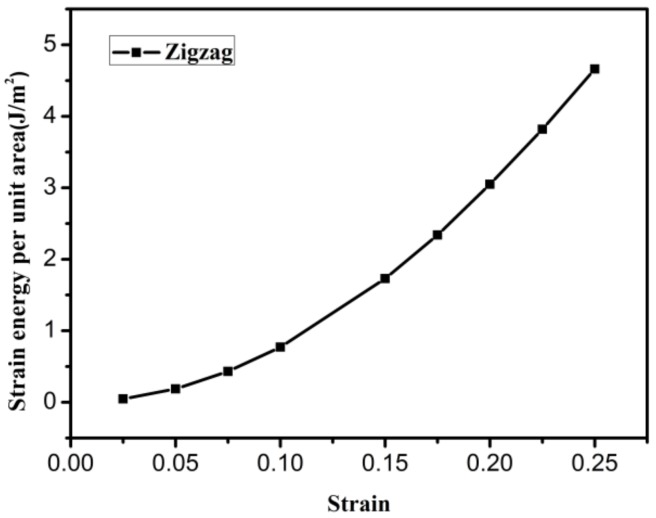
The strain energy per unit area of pristine graphene.

In this section, the initial fracture site of graphene is explored. The strain energy per unit area of pristine zigzag graphene was obtained via Equation (12). Figure 5 shows the variation in the strain energy per unit area of pristine graphene with increasing strain. The strain energy per unit area increases with increasing strain, and it will have a maximum value when the strain of the pristine graphene reaches the failure strain. The strain and strain energy per unit area were 24.6% and 4.66 J/m^2^, respectively. The results of this paper are in good agreement with those in the literature [33].

**Figure 6:**
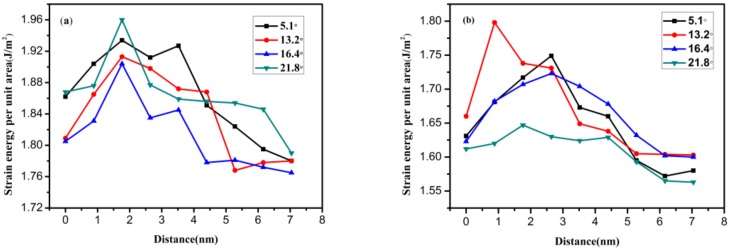
The variation in strain energy per unit area with increasing distance from the GB: (**a**) when the load is perpendicular to the GB; (**b**) when the load is parallel to the GB.

The strain energy per unit area of different graphene nanoribbons was investigated through the model in Figure 3, and then the initial fracture site of the polycrystalline graphene was analyzed. The variation in strain energy per unit area with increasing distance from the GB for different load directions (perpendicular and parallel directions to GB) is illustrated in Figure 6a,b, where the strain of polycrystalline graphene was maintained at 15%. It can be seen from these figures that the strain energy per unit area exhibits an increasing–decreasing trend with the increasing distance from the GB. These curves show that the highest strain energy per unit area is not in the position of the GB but appears in the vicinity of the GB (at a distance of about 2 nm). Furthermore, it can be inferred from these figures that when the distance from the GB is far enough, the strain energy per unit area will reduce to that of pristine graphene. The initial fracture site of polycrystalline graphene appears in the vicinity of the GB. The influence of the GB on the surrounding monocrystalline region will gradually weaken as the distance from the GB increases. This effect can be neglected when the distance is large enough.

### 3.2. The Critical Stress Intensity Factor in Pre-Cracked Monocrystalline Graphene

How is the fracture toughness affected by the GB? To answer this question, the influence of the misorientation angle and the distance between the crack tip and GB on the mode I fracture toughness will be discussed in this paper. It is well known that graphene is a nanoscale material. Its fracture process is different from that of macroscopic material. The fracture toughness of graphene could be affected by many factors such as the crack width, the crack proportion, etc. Thus, the influence of crack width and crack proportion on the mode I fracture toughness and the size effects of mode I fracture toughness were investigated in pre-cracked monocrystalline graphene. Further, the mode I fracture toughness can be expressed by the mode I critical stress intensity factor, and the parameters of the polycrystalline graphene model can be determined through the analysis of the mode I critical stress intensity factor in monocrystalline graphene.

**Figure 7:**
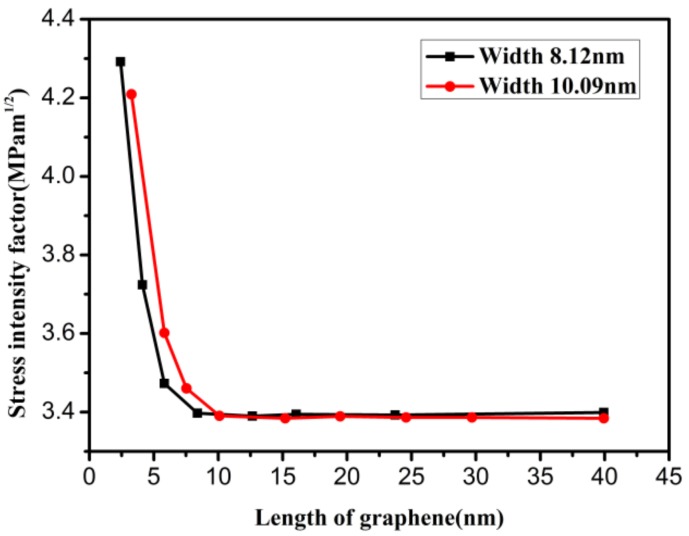
The variations of mode I critical stress intensity factor with increasing graphene length.

The size-dependent mode I critical stress intensity factor (SIF) was studied using the model in Figure 4a. Figure 7 shows the variation in the mode I critical SIF with increasing graphene length when the graphene width (the loaded edge) is fixed and the crack proportion is *f* = 0.3. It can be seen from this figure that the mode I critical SIFs of the two different graphene models have similar variation tendencies. When the graphene length is smaller than the width, the mode I critical SIF decreases sharply as the graphene length increases. However, the mode I critical SIF hardly changes with increasing graphene length when the graphene length is larger than the width. Moreover, it is interesting that the turning point in the variation trend of mode I critical SIF can be obtained when the graphene length is equal to the width. Thus, it can be inferred that there is a size effect of mode I fracture toughness, and the graphene length has little influence on the mode I critical SIF when the graphene length is larger than the width.

**Figure 8:**
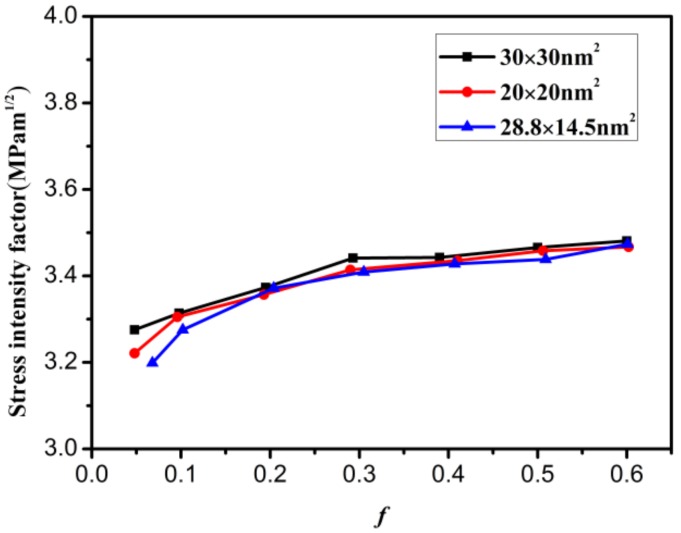
The influence of crack proportion *f* on the mode I critical stress intensity factor in different graphene models.

Two types of graphene models were created to study the effect of crack proportion *f* on the mode I critical SIF, as shown in Figure 4a. One type is a square model; the other is a rectangular model (length is larger than width). The area of the rectangular model is 400 nm^2^ and those of the square models are 400 nm^2^ and 900 nm^2^. The variation in the mode I critical SIF with increasing crack proportion *f* is shown in Figure 8. It can be seen from this figure that as the crack proportion *f* increases, the mode I critical SIFs of the three graphene models first increase and then tend to stabilize. The sizes of the three mode I critical SIFs are basically the same. In addition, the curvilinear variation tendencies show that the mode I critical SIF of graphene almost becomes a constant value when the crack proportion *f* is between 0.2 and 0.6. The range of the SIF then is 3.4–3.5 MPam^1/2^. These results are consistent with those in the literature [34,35]. From the above analysis, it can be deduced that the SIF of graphene is hardly affected when the crack proportion *f* has a reasonable range (*f* = 0.2–0.6).

**Figure 9:**
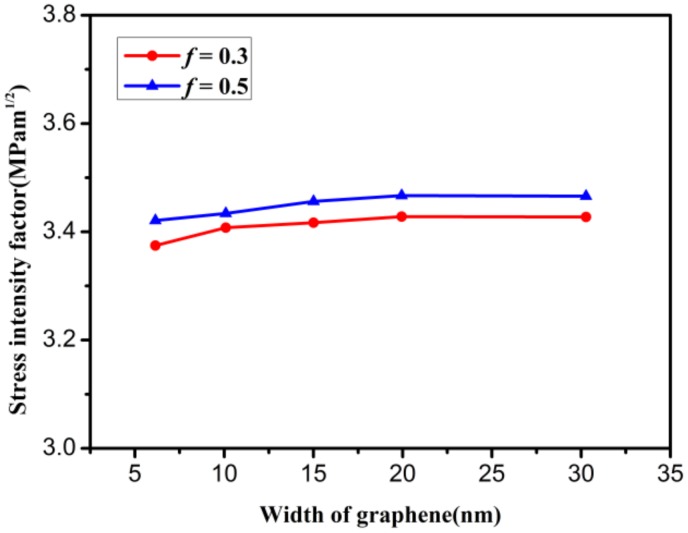
The variation in stress intensity factor with increasing graphene width.

The effect of graphene width on the mode I critical SIF is also discussed in the graphene model (as shown in Figure 4a). The absolute length of the crack was increased as the graphene width increased to ensure that the crack proportion *f* was a constant value. Figure 9 shows the variation in the mode I critical SIF with increasing graphene width when the graphene model has length *L* = 30 nm and different crack proportions *f*. It can be seen that as the graphene width increases, two types of mode I critical SIF are almost a constant value. For a graphene model with a fixed width, the changes in the mode I critical SIFs can be neglected. The SIF in Figure 9 agrees with that in Figure 8. Furthermore, it can be inferred that the mode I critical SIF of graphene has a more stable trend when the absolute length of the crack is on the order of 10 nm. The minimum length of crack in this paper is consistent with that in the literature [29].

**Figure 10:**
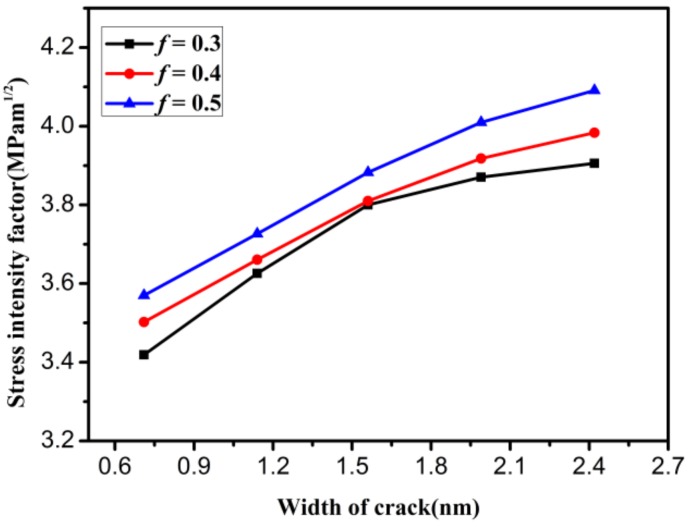
The influence of crack width on the mode I critical stress intensity factor.

In the process of graphene fracture, there is a crack-tip blunting effect. It is difficult to observe the crack-tip blunting effect in experiments, and numerical simulations are thus one of the best methods to investigate the crack-tip blunting effect of graphene. From previous numerical simulations [19,24], it is known that the crack-tip blunting effect has a significant influence on the fracture toughness. The SIF of graphene increases with increasing crack tip radius. A larger crack tip radius corresponds to larger crack width. For different crack widths, how can we obtain more realistic fracture toughness? In order to study this problem, the effect of crack width on the mode I critical SIF is discussed in a pre-cracked graphene model (shown in Figure 4a, and the size of the model is 30 × 30 nm^2^), as shown in Figure 10. It can be seen from this figure that as the crack width increases, the mode I critical SIF first increases and then gradually stabilizes. In addition, the SIF for crack proportion *f* = 0.3 has a more reasonable range when the crack width is *B ≥* 1.6 nm.

Based on all the above analysis, the other influence factors can be eliminated when the effect of GB on the mode I fracture toughness is investigated. Therefore, in this research, the size of polycrystalline graphene model and the crack length and width are 30 × 30 nm^2^, 10 nm, and 1.6 nm, respectively, as shown in Figure 4b.

### 3.3. The Influence of GB on Mode I Fracture Toughness

**Figure 11:**
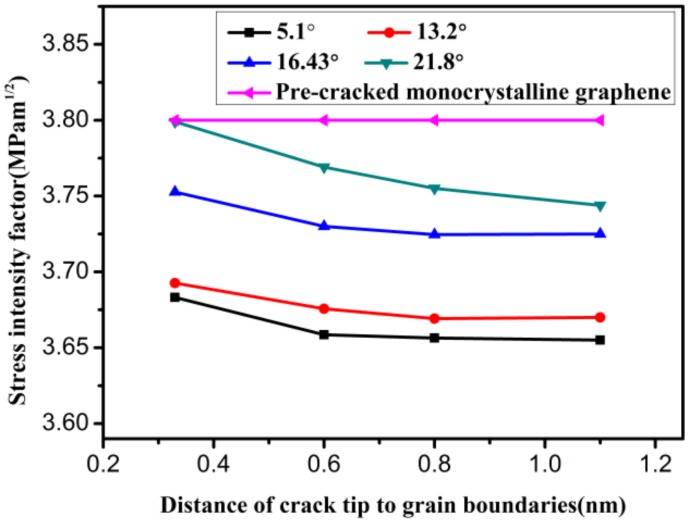
The mode I critical stress intensity factor of polycrystalline graphene with increasing distance between the crack tip and GB.

The model in Figure 4b was used to study the effect of GB on the mode I fracture toughness. Figure 11 exhibits the variation in mode I critical SIF with increasing distance between the crack tip and GB when the graphene model has different misorientation angles. It can be seen from the figure that the mode I fracture toughness of the pre-cracked polycrystalline graphene is less than that of pre-cracked pristine graphene due to the existence of a GB. As the distance between the crack tip and GB increases, the mode I critical SIF first decreases and then tends to stabilize. This is due to the GB having an effect on the surrounding monocrystalline region. Moreover, for polycrystalline graphene with different misorientation angles, the variation trend of the mode I critical SIF is different. When the misorientation angle is 21.8°, the variation trend of the mode I critical SIF is the most obvious, and the mode I critical SIF gradually reaches the level of that of the pre-cracked pristine graphene as the distance between the crack tip and GB decreases.

**Figure 12:**
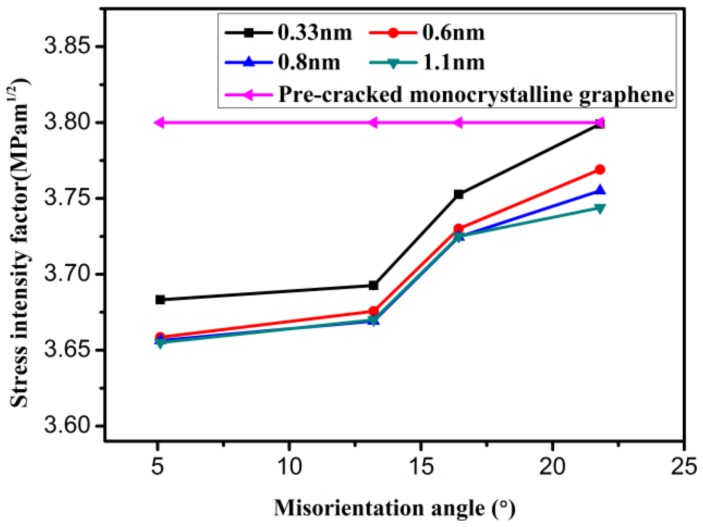
The variation in the mode I critical stress intensity factor as the misorientation angle increases.

Figure 12 illustrates the variation in the mode I critical SIF with increasing misorientation angle when the distance between the crack tip and GB is different. It can be seen from this figure that the mode I critical SIF increases with increasing misorientation angle. For different distances between the crack tip and GB, the variation tendencies of all types of mode I critical SIFs are basically the same, and the sizes of the three types of mode I critical SIF are almost consistent. Through the above analysis, it can be inferred that GBs have an impact on the mode I fracture toughness. However, the affected region is limited. The GB has an obvious influence on mode I fracture toughness when the distance between the crack tip and GB is very small. The influence of the GB will gradually weaken with increasing distance between the crack tip and GB. When the distance is large enough, the effect of the GB can be neglected.

## 4. Conclusions

In this paper, the mode I fracture toughness of pre-cracked monocrystalline/polycrystalline graphene was investigated via molecular structure mechanics and the Griffith criterion. The initial fracture site (near grain boundary) of polycrystalline graphene was revealed by the strain energy per unit area.

For monocrystalline graphene, it was found that the crack proportion and crack width have a significant influence on the mode I fracture toughness. The mode I fracture toughness has size effects. The mode I critical stress intensity factor (SIF) of graphene decreases sharply with increasing graphene length when the graphene length is less than the width (the loaded edge). However, the mode I critical SIF almost becomes a constant value when the graphene length is larger than the width. Moreover, the mode I critical SIF of graphene hardly changes when the crack proportion is between 0.2 and 0.6.

For polycrystalline graphene, the initial fracture site was discussed in terms of the strain energy per unit area. It was inferred that the initial fracture site appears in the vicinity of the GB. In addition, the conclusion was obtained that the GB has an influence on the mode I fracture toughness, but the affected region is limited. The mode I fracture toughness of pre-cracked polycrystalline graphene is less than that of pre-cracked pristine graphene due to the existence of the GB. The mode I critical SIF of pre-cracked polycrystalline graphene increases with increasing misorientation angle. However, as the distance between the crack tip and GB increases, the mode I critical SIF first decreases and then gradually stabilizes.

## Figures and Tables

**Table 1:** The parameters of the rectangular beam element [26].

b(nm)	h(nm)	$A({\mathrm{nm}}^{2})$	$I_{y}(nm^{4})$	$I_{z}(nm^{4})$	E(TPa)	G(TPa)	$J(nm^{4})$
0.1049	0.0678	$7.11\times 10^{-3}$	$2.73\times 10^{-6}$	$6.53\times 10^{-6}$	14.56	2.30	$9.26\times 10^{-6}$

**Table 2:** The parameters of the modified Morse potential [27,28].

$r_{0}(m)$	$\beta (m^{-1})$	$D_{e}(N\cdot m)$	$\theta_{0}(\mathrm{rad})$	$k_{\theta }(N\cdot m/rad^{2})$	$k_{sextic}(rad^{-4})$
$1.421\times 10^{-10}$	$2.625\times 10^{10}$	$6.03105\times 10^{-19}$	2.094	$0.9\times 10^{-18}$	0.754

## References

[1] Wang Y., Liu Z. (2016). The fracture toughness of graphene during the tearing process. Model. Simul. Mater. Sci. Eng..

[2] Dikin D.A., Stankovich S., Zimney E.J., Piner R.D., Dommett G.H.B., Evmenenko G., Nguyen S.T., Ruoff R.S. (2007). Preparation and characterization of graphene oxide paper. Nature.

[3] Potts J.R., Lee S.H., Alam T.M., An J., Stoller M.D., Piner R.D., Ruoff R.S. (2011). Thermomechanical properties of chemically modified graphene/poly (methyl methacrylate) composites made by in situ polymerization. Carbon.

[4] Bunch J.S., Van Der Zande A.M., Verbridge S.S., Frank I.W., Tanenbaum D.M., Parpia J.M., Craighead H.G., McEuen P.L. (2007). Electromechanical resonators from graphene sheets. Science.

[5] Singh V., Sengupta S., Solanki H.S., Dhall R., Allain A., Dhara S., Pant P., Deshmukh M.M. (2010). Probing thermal expansion of graphene and modal dispersion at low-temperature using graphene nanoelectromechanical systems resonators. Nanotechnology.

[6] Li X., Cai W., An J., Kim S., Nah J., Yang D., Piner R., Velamakanni A., Jung I., Tutuc E. (2009). Large-area synthesis of high-quality and uniform graphene films on copper foils. Science.

[7] Reina A., Jia X., Ho J., Nezich D., Son H., Bulovic V., Dresselhaus M.S., Kong J. (2008). Large area, few-layer graphene films on arbitrary substrates by chemical vapor deposition. Nano Lett..

[8] An J., Voelkl E., Suk J.W., Li X., Magnuson C.W., Fu L., Tiemeijer P., Bischoff M., Freitag B., Popova E. (2011). Domain (grain) boundaries and evidence of “twinlike” structures in chemically vapor deposited grown graphene. ACS Nano.

[9] Liu T.H., Pao C.W., Chang C.C. (2012). Effects of dislocation densities and distributions on graphene grain boundary failure strengths from atomistic simulations. Carbon.

[10] Kim K., Lee Z., Regan W., Kisielowski C., Crommie M.F., Zettl A. (2011). Grain boundary mapping in polycrystalline graphene. ACS Nano.

[11] Huang P.Y., Ruiz-Vargas C.S., van der Zande A.M., Whitney W.S., Levendorf M.P., Kevek J.W., Garg S., Alden J.S., Hustedt C.J., Zhu Y. (2011). Grains and grain boundaries in single-layer graphene atomic patchwork quilts. Nature.

[12] Ruiz-Vargas C.S., Zhuang H.L., Huang P.Y., van der Zande A.M., Garg S., McEuen P.L., Muller D.A., Hennig R.G., Park J. (2011). Softened elastic response and unzipping in chemical vapor deposition graphene membranes. Nano Lett..

[13] Grantab R., Shenoy V.B., Ruoff R.S. (2010). Anomalous strength characteristics of tilt grain boundaries in graphene. Science.

[14] Wei Y., Wu J., Yin H., Shi X., Yang R., Dresselhaus M. (2012). The nature of strength enhancement and weakening by pentagon–heptagon defects in graphene. Nat. Mater..

[15] Xu N., Guo J.G., Cui Z. (2016). The influence of tilt grain boundaries on the mechanical properties of bicrystalline graphene nanoribbons. Phys. E Low-Dimens. Syst. Nanostruct..

[16] Wu J., Wei Y. (2013). Grain misorientation and grain-boundary rotation dependent mechanical properties in polycrystalline graphene. J. Mech. Phys. Solids.

[17] Zhang J., Zhao J., Lu J. (2012). Intrinsic strength and failure behaviors of graphene grain boundaries. ACS Nano.

[18] Yi L., Yin Z., Zhang Y., Chang T. (2013). A theoretical evaluation of the temperature and strain-rate dependent fracture strength of tilt grain boundaries in graphene. Carbon.

[19] Zhang P., Ma L., Fan F., Zeng Z., Peng C., Loya P.E., Liu Z., Gong Y., Zhang J., Zhang X. (2014). Fracture toughness of graphene. Nat. Commun..

[20] Aliha M.R.M., Bahmani A., Akhondi S. (2015). Determination of mode III fracture toughness for different materials using a new designed test configuration. Mater. Des..

[21] Bahmani A., Aliha M.R.M., Berto F. (2017). Investigation of fracture toughness for a polycrystalline graphite under combined tensile-tear deformation. Theor. Appl. Fract. Mech..

[22] Jung G.S., Qin Z., Buehler M.J. (2015). Molecular mechanics of polycrystalline graphene with enhanced fracture toughness. Extrem. Mech. Lett..

[23] Shekhawat A., Ritchie R.O. (2016). Toughness and strength of nanocrystalline graphene. Nat. Commun..

[24] Han J., Sohn D., Woo W., Kim D.K. (2017). Molecular dynamics study of fracture toughness and trans-intergranular transition in bi-crystalline graphene. Comput. Mater. Sci..

[25] Li C., Chou T.W. (2003). A structural mechanics approach for the analysis of carbon nanotubes. Int. J. Solids Struct..

[26] Li H., Guo W. (2008). Transversely isotropic elastic properties of single-walled carbon nanotubes by a rectangular beam model for the C-C bonds. J. Appl. Phys..

[27] Tserpes K.I., Papanikos P. (2007). The effect of Stone–Wales defect on the tensile behavior and fracture of single-walled carbon nanotubes. Compos. Struct..

[28] Belytschko T., Xiao S.P., Schatz G.C., Ruoff R.S. (2002). Atomistic simulations of nanotube fracture. Phys. Rev. B.

[29] Yin H., Qi H.J., Fan F., Zhu T., Wang B., Wei Y. (2015). Griffith criterion for brittle fracture in graphene. Nano Lett..

[30] Bazant Z.P., Planas J. (1997). Fracture and Size Effect in Concrete and Other Quasibrittle Materials.

[31] Griffith A.A. (1921). The phenomena of rapture and flow in solids. Philos. Trans. R. Soc. Lond. A.

[32] Ayatollahi M.R., Akbardoost J., Berto F. (2016). Size effects on mixed-mode fracture behavior of polygranular graphite. Carbon.

[33] Le M.Q., Batra R.C. (2016). Mode-I stress intensity factor in single layer graphene sheets. Comput. Mater. Sci..

[34] Zhang B., Mei L., Xiao H. (2012). Nanofracture in graphene under complex mechanical stresses. Appl. Phys. Lett..

[35] Datta D., Nadimpalli S.P.V., Li Y., Shenoy V.B. (2015). Effect of crack length and orientation on the mixed-mode fracture behavior of graphene. Extrem. Mech. Lett..

